# Age-related differences in neuroinflammatory responses associated with a distinct profile of regulatory markers on neonatal microglia

**DOI:** 10.1186/1742-2094-11-70

**Published:** 2014-04-04

**Authors:** Leah B Christensen, Tyson A Woods, Aaron B Carmody, Byron Caughey, Karin E Peterson

**Affiliations:** 1 Laboratory of Persistent Viral Diseases, Rocky Mountain Laboratories, National Institute of Allergy and Infectious Diseases, National Institutes of Health, Hamilton MT 59840, USA

## Abstract

**Background:**

The perinatal period is one in which the mammalian brain is particularly vulnerable to immune-mediated damage. Early inflammation in the central nervous system (CNS) is linked with long-term impairment in learning and behavior, necessitating a better understanding of mediators of neuroinflammation. We therefore directly examined how age affected neuroinflammatory responses to pathogenic stimuli.

**Methods:**

In mice, susceptibility to neurological damage changes dramatically during the first few weeks of life. Accordingly, we compared neuroinflammatory responses to pathogen associated molecular patterns (PAMPs) of neonatal (two day-old) and weanling (21 day-old) mice. Mice were inoculated intracerebrally with PAMPs and the cellular and molecular changes in the neuroinflammatory response were examined.

**Results:**

Of the 12 cytokines detected in the CNS following toll-like receptor 4 (TLR4) stimulation, ten were significantly higher in neonates compared with weanling mice. A similar pattern of increased cytokines in neonates was also observed with TLR9 stimulation. Analysis of cellular responses indicated a difference in microglial activation markers in the CNS of neonatal mice and increased expression of proteins known to modulate cellular activation including CD11a, F4/80 and CD172a. We also identified a new marker on microglia, SLAMF7, which was expressed at higher levels in neonates compared with weanlings.

**Conclusions:**

A unique neuroinflammatory profile, including higher expression of several proinflammatory cytokines and differential expression of microglial markers, was observed in brain tissue from neonates following TLR stimulation. This increased neuroinflammatory response to PAMPs may explain why the developing brain is particularly sensitive to infection and why infection or stress during this time can lead to long-term damage in the CNS.

## Background

During the perinatal period, the mammalian brain is developing rapidly and is particularly sensitive to inflammation or maternal stress. Gestational viral, bacterial and parasitic infections have been linked to neurological illnesses in offspring, including cerebral palsy and schizophrenia [1-3]. Additionally, perinatal infection is a factor for developing neurodegenerative diseases such as Alzheimer’s and Parkinson’s later in life [4-6]. Current research suggests that it may not be the infectious agent *per se* but the immune response that is causing the neurological damage [2,7,8]. One useful model is the neonatal mouse, where cortical development roughly corresponds to the human fetus midway through gestation [9,10]. Neonatal rodent models have demonstrated that perinatal immune stimulation with either infectious agents or Toll-like receptor (TLR) ligands can produce developmental and behavioral changes similar to those observed in human neurological illnesses, including alterations in learning and memory [8,11]. Understanding the immune response in the CNS during the perinatal period is necessary in order to understand how developmental abnormalities and neurological damage may occur.

Studies of immune cells in neonates indicate that the peripheral immune response is often suppressed in response to infection or immune stimuli [12-15]. As such, neonates and infants are more susceptible to viral and bacterial infections compared with adults [12,16,17]. This suppression may be due in part to reduced activation of neonatal monocytes to TLR activation compared with adult monocytes [13-15,18,19]. Inability to respond to viral or bacterial infections may allow infections to spread and persist in the neonatal host, resulting in increased damage to healthy tissues compared with adults.

It is unclear whether the suppressed immune response observed in the periphery of neonates during development is also observed in the CNS. During development, microglial cells, which are considered to be the resident macrophage population of the brain, are in an active state [20-22]. Microglia are derived from the yolk sac and migrate into the CNS during the perinatal period where they are actively involved in pruning synapses from neurons [22,23]. Following this process, microglia undergo a ramification process where they become quiescent and persist in this state unless activated by insult or injury [20]. Thus, microglia may actually be more activated, and thus possibly more responsive to immune stimuli, during the perinatal/neonatal period compared with adults.

Here we have examined neuroinflammatory responses in neonatal and weanling mice by inoculating them with two pathogen associated molecular patterns (PAMPs) that have been used for modeling neurodevelopmental illness: lipopolysaccharide (LPS), the ligand for TLR4, and unmethylated CpG oligodeoxynucleotides (CpG-ODN), the ligand for TLR9. Increased expression of proinflammatory cytokines and chemokines, as well as other neuroinflammatory markers, were significantly elevated in the brains of neonatal mice compared with weanling mice. We then examined the myeloid cell population within the CNS to examine possible mediators of the differential inflammatory response.

## Methods

### Ethics statement

All animal research was carried out in adherence with protocols approved by the National Institutes of Health Rocky Mountain Laboratories Animal Care and Use Committee with animal protocols 2008-46 and 2009-70.

### Animal models and inoculation of TLR agonists

All mice were housed and maintained by the Rocky Mountain Laboratories Veterinary Branch (Hamilton, MT, USA). Mice were maintained under pathogen-free conditions with regular light/dark cycles and given food and water *ad libitum*. For inoculations, neonatal (two day-old) C57BL/10 mice were anaesthetized by hypothermia, while weanling (21 day-old) mice were anaesthetized by isofluorane inhalation prior to inoculation. Mice were inoculated intracerebrally (ic), using a Hamilton syringe with a 33-gauge needle, once in each hemisphere with a solution volume of 3 μl per hemisphere. Mice were inoculated with either LPS, CpG-ODN or as an inoculation control, PBS. We used LPS and CpG-ODN concentrations that, based on preliminary studies, were expected to elicit strong TLR-mediated responses without being lethal. Mice were inoculated with either 0.5 μg LPS per gram of body weight or 0.125 μg CpG-ODN per gram of body weight. For neonatal mice, this meant a total inoculum of 1 μg of LPS or 0.25 μg (40 picomoles) of CpG-ODN. For three week old mice, this translated to 3.8 μg of LPS or 0.95 μg of CpG-ODN. Uninoculated mice were also used as additional controls. To control for any possible variations between litters, agonist preparation or inoculations, all experimental groups contained mice from different litters that were inoculated on different days.

At the indicated time points, all animals were anaesthetized by inhalation of isofluorane and euthanized using axillary incision. Brain tissue was snap frozen in liquid nitrogen and stored at −80°C until use.

### TLR agonists

TLR4 agonist ultra-pure LPS (catalog number tlrl-3pelps) and TLR9 agonist phosphorothioated CpG-ODN type B [5′-tcc atg acg ttc ctg acg tt-3′] (catalog number tlrl-1826) were purchased from InvivoGen (San Diego, CA, USA). Agonist stocks were suspended in endotoxin-free water, aliquoted and stored at −20°C. Immediately prior to use, agonists were diluted in endotoxin-free, PBS-buffered solution.

### Protein quantification

For protein quantification, brain tissue was weighed and homogenized in Bio-Plex cell lysis buffer (Bio-Rad, Hercules, CA, USA; catalog number 171-304012) containing PMSF (Sigma, St. Louis, MO, USA; catalog number P-7626) and Complete Mini Protease Inhibitor Cocktail (Roche, Basel, CH; catalog number 11836153001) as previously described [24]. All samples were diluted to a final concentration of 300 mg/ml of brain tissue in lysis buffer. Samples were then centrifuged at 4,500 × *g* for 15 minutes at 4°C to remove debris. Cytokine and chemokine protein levels in brain homogenate supernatants were analyzed using the Invitrogen Mouse Cytokine Twenty-Plex Antibody Bead kit (Carlsbad, CA, USA; catalog number LMC006) on a Bio-Rad Bio-Plex 200 system (Hercules, CA, USA). Individual protein concentrations were calculated using standard curves generated from standards provided with the Twenty-Plex kit.

### Quantification of mRNA expression by real-time PCR

Total RNA was extracted from brain tissue using the Qiagen RNeasy Mini Kit (Valencia, CA, USA; catalog number 74106) per the manufacturer’s instructions. RNA was then treated with DNase (Ambion, Foster City, CA, USA; catalog number AM2224) for 30 minutes at 37°C, followed by a final purification and concentration using the Zymo Research RNA Clean-up kit (catalog number R1018). Complimentary DNA (cDNA) was generated from the isolated RNA using the iScript cDNA Synthesis kit (Bio-Rad, Hercules, CA, USA; catalog number 170-8891). All primers were designed using Primer3 and were gene-specific in blast searches performed using the National Center for Biotechnology Information database as previously described [24]. Real-time PCR was performed using iTAQ SYBR Green Supermix with ROX (Bio-Rad, Hercules, CA, USA, catalog number 1725852) on an Applied Biosystems (Grand Island, NY, USA) PRISM 7900HT instrument. All samples were run in triplicate. The baseline was automatically set and the C_T_ was manually set to intersect the mid-log phase of PCR curves at 0.19. Dissociation curves were used to verify that only a single gene product was amplified in each sample. Data analysis was completed following the published criteria for real-time quantitative PCR method using the comparative C_T_ method [25,26]. Gene expression was calculated relative to the average expression of three housekeeping genes (Actb, Gusb, Rpl32) for each sample within an experiment. The same housekeeping genes were used for each experiment. Data are shown as the percent of the average of the housekeeping genes (% of housekeeping genes). RNA that was not reverse transcribed and water were used as negative controls.

### Preparation of brain tissue for flow cytometry

Animals were anaesthetized by inhalation of isofluorane, followed by perfusion through the left ventricle of the heart with ice cold 1× Hank’s balanced salt solution (HBSS) without calcium and magnesium (Gibco, Grand Island, NY, USA; catalog number 14185). Whole brains were removed and sliced into several pieces prior to homogenization with a Dounce homogenizer and/or trituration using a 5 ml pipet. Samples were further triturated using a 20-gauge needle. For some experiments, brain homogenates were further dissociated by incubation in 0.05% Collagenase D (Roche, Basel, CH; catalog number 11 088 882 001), 0.09 U/ml Dispase I (Sigma, St. Louis, MO, USA; catalog number D4818) and 0.025 U/ml DNase I (Sigma, catalog number D4527) in 1× HBSS at room temperature for 30 minutes with continuous rocking. To isolate the myeloid and immune cells, cells were resuspended in 70% Percoll/HBSS and fractionated using a Percoll gradient as previously described [27]. Cells were harvested from the 30/70% interface of the Percoll gradient, washed in HBSS and analyzed by flow cytometry or used for quantitative real-time PCR.

### Flow cytometric analysis of CNS populations *ex vivo*

Following isolation by Percoll gradient, samples were plated onto 96-well plates and analyzed for cell surface markers as previously described [28]. In short, cells were fixed in 2% paraformaldehyde, then permeabilized in 0.1% saponin/2% BSA/1× PBS. Samples were incubated in an F_C_ blocking solution containing rat anti-mouse CD16/CD32 Fcγ III/II antibody (BD Pharmingen, San Diego, CA, USA; catalog number 553142) in 2% donkey serum/0.1% saponin/2% BSA/1× PBS. Cells were incubated with fluorochrome-conjugated antibodies at room temperature. After washing twice, cells were resuspended in PBS and analyzed on a FACSAria flow cytometer (BD Biosciences, San Jose, CA, USA) using FACSDiva software (BD Biosciences, San Jose, CA, USA). Cells were gated as described in the figure legends.

## Results

### Neuroinflammatory responses are increased in neonates compared with weanling mice

To determine if the neuroinflammatory response changes in the first weeks of life, we inoculated neonatal (two day-old) and weanling (21 day-old) mice ic with either the TLR4 agonist, LPS, or the TLR9 agonist, CpG-ODNs. Control mice were inoculated with vehicle controls.

**Figure 1:**
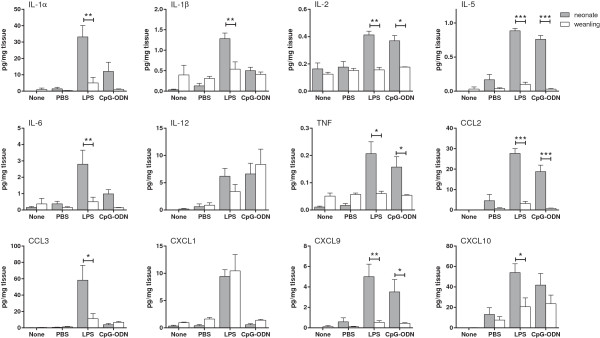
**Heightened neonatal cytokine responses in the CNS following TLR4 and TLR9 stimulation.** Neonatal (gray bars) and weanling (open bars) mice were inoculated intracerebrally (ic) with LPS or CpG-ODN at the concentrations described in the Methods section. Control groups were either not inoculated (none) or inoculated ic with PBS. At 12 hpi, brain tissue was removed and processed for protein analysis. Cytokine protein levels in brain homogenates from neonatal and weanling mice were measured using a Multiplex bead assay. n = 10 to 12 for neonates and 5 to 6 for weanling mice. Data are presented as mean ± SEM. Statistical analysis was completed by two-way analysis of variance with Sidak’s multiple comparisons test. Significant age-specific differences are as indicated: *P < 0.05, **P < 0.01, ***P < 0.001.

Cytokine protein levels were examined at 12 hours post inoculation (hpi), a time point previously shown to be the peak of cytokine production following TLR ligand inoculation in the CNS [29,30]. Twelve cytokines were detectable at significant levels following TLR stimulation of the CNS. Several of these cytokines, including IL-1α, IL-1β, IL-2, IL-5, IL-6, TNF, *CC* chemokine ligand (CCL)2, CCL3 and CXCL9 were significantly higher in neonatal brain tissue than in weanling brain tissue in response to LPS (Figure 1). CpG-ODN also induced higher responses in neonates than in weanling mice for IL-2, IL-5, TNF, CCL2 and CXCL9. No significant age-dependent differences were observed for IL-12 or CXCL1. The cytokines tested were undetectable at later time points of 24, 48 or 72 hours for both neonates and weanling mice. Overall, the levels of several cytokines induced by TLR4 and/or TLR9 ligand induction were significantly higher in neonates than those observed in weanlings.

**Figure 2:**
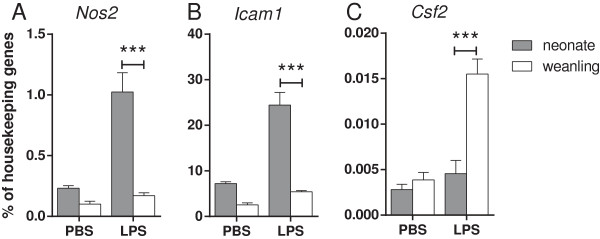
**Increased mRNA expression of inflammatory markers in neonatal brain tissue following LPS stimulation.** Real-time PCR analysis was used to analyze the expression of neuroinflammatory markers in brain tissue from neonatal and weanling mice at 6 hpi. Increased mRNA expression of *Nos2* (iNos) **(A)** and *Icam1***(B)** mRNAs were observed in brain tissue from LPS-inoculated neonatal mice, while *Csf2* (GMCSF) mRNA **(C)** was elevated in brain tissue from LPS-inoculated weanling mice. n = 4 mice per group. Data are presented as mean ± SEM. Statistical analysis was completed by two-way analysis of variance with Tukey’s multiple comparisons test. Significant age-specific differences in the LPS response are indicated in the figure as: ***P < 0.001.

Since a difference in the expression of several cytokines was observed between weanling and neonates, we further examined neuroinflammatory responses using real-time PCR analysis. RNA was analyzed at 6 hpi, a timepoint that correlates with peak mRNA expression of cytokines following TLR stimulation both *in vitro* and *in vivo*[28,30]. Analysis of mRNA expression also indicated similarly increased levels of several of these cytokines in neonatal mice as well as increased expression of inducible nitric oxide (*Nos2*, *iNos*) mRNA and adhesion molecule *Icam1* mRNA (Figure 2, data not shown). In contrast, expression of *Csf2* (GM-CSF) mRNA, a growth factor for monocytes, was elevated in brain tissue from weanling mice compared with neonatal mice (Figure 2). Thus, LPS inoculation induced different neuroinflammatory responses in neonates compared with weanlings with increased production of several cytokines and decreased expression of *Csf2* mRNA.

**Figure 3:**
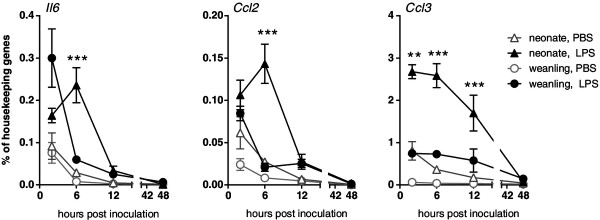
**Time course analyses of cytokine mRNA expression indicate differences in the brain cytokine response between neonates and weanlings.** Cytokine mRNA levels in brains from neonatal and weanling mice were assayed at 2, 6, 12 and 48 hpi following ic inoculation with PBS or LPS. n = 4 to 6 mice per group per time point from combined experiments. Data are presented as mean ± SEM. Statistical analysis was completed by two-way analysis of variance with Tukey’s multiple comparisons test. Significant age-specific differences in the LPS response between neonates and weanlings are indicated: **P < 0.01, ***P < 0.001.

To examine the time course of these neuroinflammatory responses, we measured mRNA expression levels from 2 to 48 hpi after treatment with either LPS or vehicle control. Modest increases in gene expression for *Ccl2* and *Ccl3* mRNA were observed in vehicle control-treated animals for both neonatal and weanling brains at 2 hpi but not at other time points. At 2 hpi after stimulation with LPS, *Ccl2* and *Il6* mRNA levels were comparable between the two ages, while *Ccl3* mRNA was higher in neonates (Figure 3). However, by 6 hpi, these LPS responses in weanling mice had dropped to nearly basal levels while neonatal *Il6* and *Ccl2* levels remained elevated (Figure 3). Thus, expression of certain cytokine mRNAs were generally higher in neonates and peak expression occurred later than that observed in weanlings. This difference in kinetics may contribute to the increased protein levels of these cytokines observed at 12 hpi (Figure 1).

**Figure 4:**
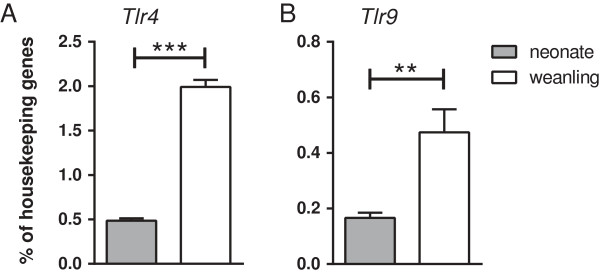
***Tlr4 *and *Tlr9 *mRNA expression is influenced by age.***Tlr4***(A)***and Tlr9***(B)** mRNA levels were measured in the brains from untreated neonatal and weanling mice. Housekeeping gene expression was averaged using *Gusb*, *Actb* and *Rpl32*. Data are presented as mean ± SEM (n = 5 to 6 per group). Statistical analysis was completed by an unpaired *t*-test. **P < 0.01, ***P < 0.001.

One possible explanation for the increased neuroinflammatory responses in neonatal mice would be higher expression of TLRs in the brains of neonates than adult mice. However, analysis of mRNA demonstrated lower levels of *Tlr4* and *Tlr9* mRNA in neonatal brain compared with other ages (Figure 4). Thus, the increased neuroinflammatory response is not likely to be due to increased expression of TLRs in the neonatal brain.

### Differences in immune cell markers between neonates and weanlings

**Figure 5:**
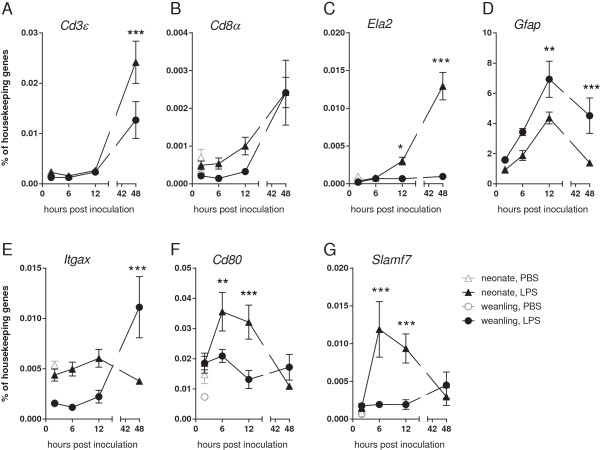
**Temporal expression of glial and immune cell markers after TLR4 stimulation.** Brains from neonatal and weanling mice (described in Figure 3) were analyzed for **(A-G)** inflammatory cell markers as well as astrocytic and myeloid cell markers. An increase in T cell markers *Cd3ϵ***(A)** and *Cd8α***(B)**, as well as neutrophil marker *Ela2***(C)** was not observed until 12 to 48 hours post infection. *Gfap***(D)** mRNA was upregulated at 6 to 12 hours post infection with higher levels in weanling mice. In contrast, myeloid markers *Itgax***(E)** and *Cd80***(F)** were higher in neonates than weanlings. *Slamf7***(G)**, which is expressed by a number of immune cells, was also elevated in neonates compared with weanlings. Black symbols indicate data from LPS-stimulated mice, while gray symbols indicate PBS-treated controls. n = 4 to 6 mice per group per time point from combined experiments. Data are presented as mean ± SEM. Statistical analysis was completed by two-way analysis of variance with Tukey’s multiple comparisons test. Significant age-specific differences in the LPS response are as indicated: *P < 0.05, **P < 0.01, ***P < 0.001.

To investigate which cell types might be contributing to the heightened neonatal inflammatory response, we compared mRNA expression levels of inflammatory cells and glial cells in brain tissue from neonatal and weanling mice. Analysis of gene expression for inflammatory cells including T cells (*Cd3ϵ*, *Cd8α*) and neutrophils (*Ela2*) showed that these genes were only induced at 12 to 48 hpi (Figure 5A-C), after the peak cytokine response (Figure 3). Thus, an influx of T cells or neutrophils would probably be responding to, rather than producing, early increases in cytokine levels. The level of mRNA for the astrocyte activation marker *Gfap* was lower in neonatal brain than weanling brains (Figure 5D), suggesting that more activated astrocytes were also not likely to be the source of heightened cytokine responses in neonates. However, mRNAs for activation markers of myeloid cells (for example, microglia and macrophages), namely *Itgax* (Cd11c) and *Cd80*, were elevated at 6 to 12 hpi in neonates compared with weanlings (Figure 5E-F), in concert with the increases in the various cytokine mRNAs. Interestingly, at these time points we also observed elevated mRNA levels for *Slamf7* (Figure 5G), whose expression has been reported on natural killer (NK) cells and other immune cells [31,32]. Thus, the only cell markers that correlated with the early neuroinflammatory response in neonates were those thought to be primarily for myeloid cells and/or NK cells.

### Myeloid cells from brain tissue of neonatal mice have heightened responses to LPS stimulation

Since resident myeloid cells and/or inflammatory cells such as NK cells could be contributing to the cytokine response, we isolated these cells from the brains of neonatal and weanling mice using a Percoll gradient. The 6 hpi time point was used to allow for RNA analysis of isolated cell fractions. We analyzed cells from the 30/70% interface, which are predominantly microglia, macrophages and infiltrating immune cells [33]. Analysis of this cell fraction for CD4+ T cells, CD8+ T cells, NK cells and B cells indicated no significant populations of these cells in the brain for either neonatal or weanling mice (data not shown). Instead, the primary cells in this fraction for both neonates and weanling mice were CD45+ CD11b + myeloid cells; primarily microglia, monocytes and resident macrophages (data not shown).

**Figure 6:**
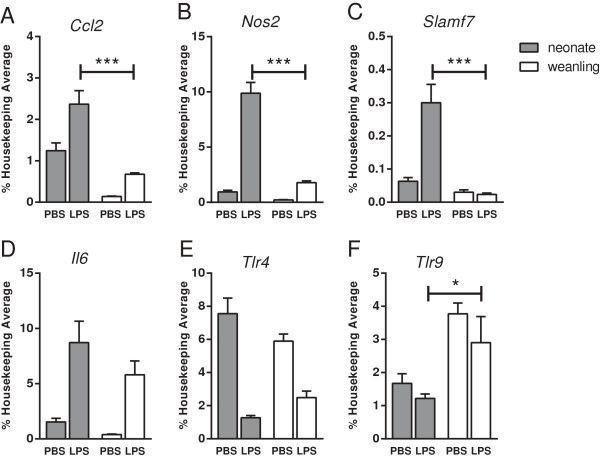
**Neonatal versus weanling expression of neuroinflammatory genes in myeloid cells isolated from the CNS.** At six hours after ic inoculation with PBS or LPS, brain homogenates from neonatal and weanling mice were fractionated on Percoll gradients, and mRNA expression levels were analyzed by qRT-PCR. mRNA expression of neuroinflammatory markers *Ccl2***(A)**, *Nos2***(B)** and *Slamf7***(C)** were elevated in the myeloid fraction from LPS-inoculated neonatal mice. *Il6***(D)** and *Tlr4***(E)** mRNA were not significantly different between age groups, while *Tlr9***(F)** mRNA levels were increased in myeloid cells from weanling mice. n = 4 to 5 mice per group from combined experiments. Data are presented as mean ± SEM. Statistical analysis was completed by two-way analysis of variance with Tukey’s multiple comparisons test. Significant age-specific-differences in the response to LPS are shown: ***P < 0.001.

RNA analysis of the myeloid cell fraction for genes analyzed in whole brain fractions demonstrated greater upregulation of *Ccl2*, *Nos2* and *Slamf7* mRNA following LPS stimulation in neonates compared with weanlings (Figure 6A-C). However, other cytokines, such as *Il6*, that were upregulated in neonates at 6 hpi in whole brain tissue (Figure 3) were not significantly different in the myeloid cell fractions at this time point (Figure 6D). Thus, myeloid cells isolated from the brain tissue of neonatal mice had increased upregulation of some of the same proinflammatory genes that were upregulated in the whole brain, indicating that these cells were likely contributing to the increased neuroinflammatory response in neonatal mice.

Interestingly, analyses of *Tlr4* and *Tlr9* mRNA expression in the myeloid cell fractions indicated no significant difference in *Tlr4* mRNA expression in the myeloid fraction from neonatal and weanling mice, while a slight increase in *Tlr9* mRNA was observed in weanlings (Figure 6E-F). Thus, the difference in the cytokine response between neonatal and weanling myeloid cells did not correlate with differences in TLR mRNA expression.

### Differences in the myeloid cell populations in the brains of neonatal and weanling mice

**Figure 7:**
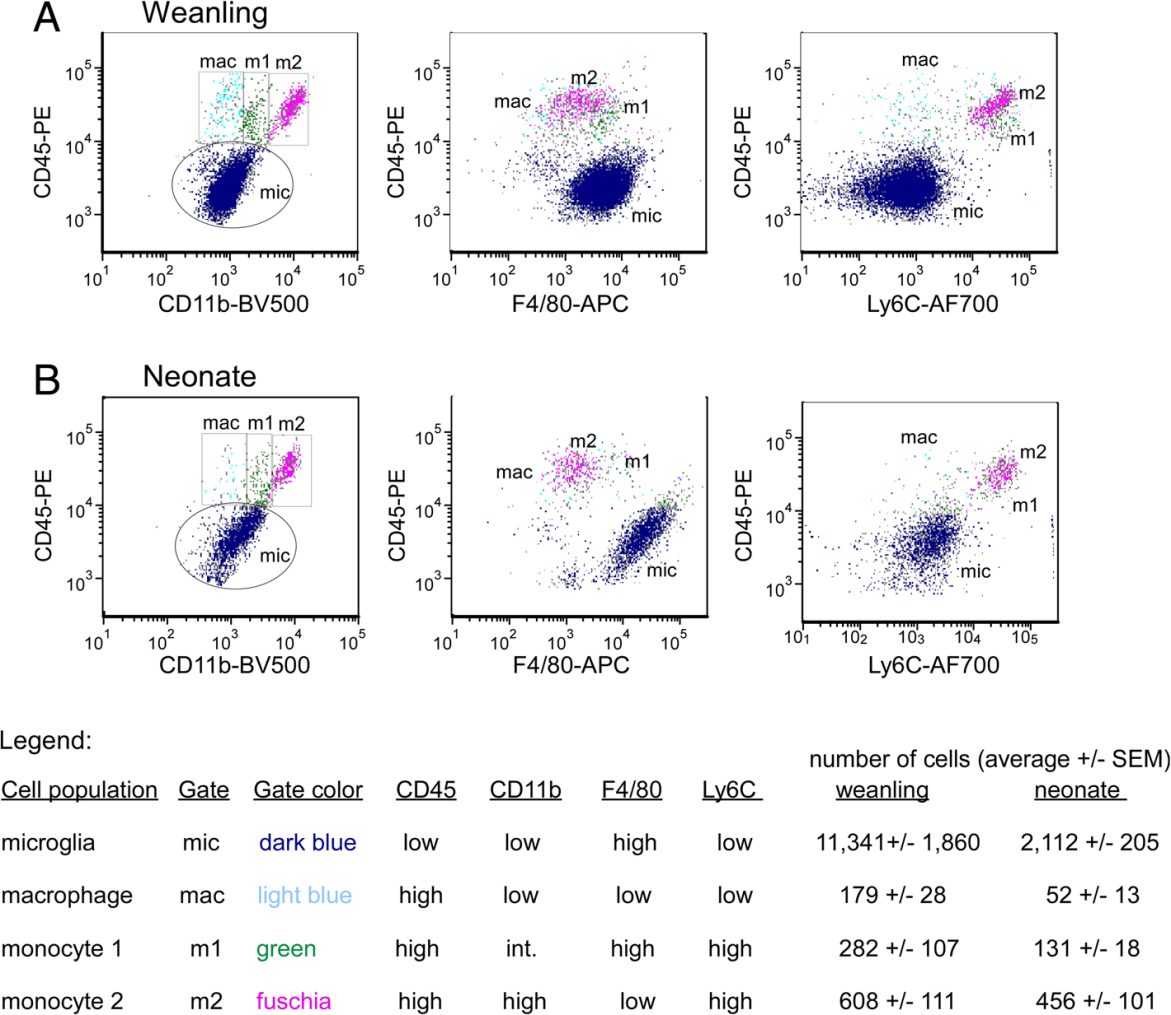
**Differences in the microglial and resident macrophage populations in the CNS of neonatal and weanling mice.** Cells from weanling **(A)** and neonatal **(B)** mice at 6 hpi with LPS were isolated on Percoll gradients and the myeloid cell fraction was collected from the 30/70% interface and analyzed by flow cytometry. An initial gate was drawn based on forward and side scatter to exclude cellular debris and doublets. This cell fraction was further gated on only CD45+ and CD11b + cells to directly analyze the myeloid cells. The CD45+ CD11b + cells were subsequently divided into subpopulations based on their relative expression of CD11b and CD45. These included a CD45^lo^, CD11b + microglial population (dark blue gate) and three populations of CD45^hi^ cells: CD11b^lo^ (light blue), CD11b^int^ (green) and CD11b^hi^ (fuchsia). These populations were then analyzed using CD45 and F4/80 or CD45 and Ly6C to identify resident macrophage and monocyte populations. Data are representative of 4 to 5 mice per group.

Since RNA analysis of myeloid cells from neonatal and weanling mice indicated differences in their neuroinflammatory responses, we further analyzed these cells by flow cytometry at 6 hpi, the time point that mRNA differences were observed. Analysis of cells expressing leukocyte common antigen CD45 and the myeloid-specific marker CD11b identified four distinct cell populations. These included CD45^lo^, CD11b^lo^ microglia (mic, dark blue gate), and three populations of CD45^hi^ cells that varied in their expression of CD11b (light blue, green and fuchsia gates) (Figure 7). Further analysis using the inflammatory monocyte marker Ly6C indicated that these three cell populations were either Ly6C + monocytes (m1 green and m2 fuchsia gates) or Ly6C^−^ resident macrophages (mac, light blue gate). The monocyte populations have two distinct profiles in the weanling mice which differed in the expression of CD11b and F4/80, being either CD11b^int^, F4/80^hi^ (m1, green gate) or CD11b^hi^ and F4/80^lo^ (m2, fuschia gate). Lower numbers of all cell populations were observed in neonates compared with weanlings; most notably, the microglia population (mic, dark blue gate) had five-fold fewer cells in neonates than weanlings (Figure 7). In addition, microglia had higher levels of F4/80 (middle panel, X axis) compared with weanlings indicating that these cells change phenotypically between these two ages. Of the CD45^hi^ populations, the resident macrophage (mac, light blue gate) was also reduced in neonates and was barely detectable.

**Figure 8:**
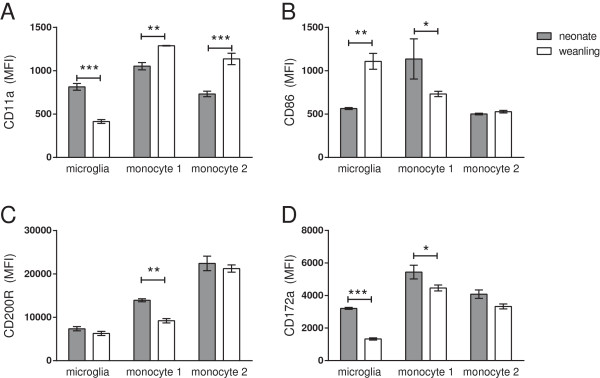
**Neonatal versus weanling expression of activating and inhibitory markers on microglia.** Cells shown in Figure 7 were additionally analyzed for expression of activation markers CD11a **(A)** and CD86 **(B)** as well as inhibitory markers CD200R **(C)** and CD172a **(D)**. Mean Fluorescent Intensity (MFI) of microglia (CD45^lo^, CD11b^lo^ dark blue gate in Figure 7) and both monocyte populations are shown. Monocyte 1 population contained cells that were CD11b^int^, F4/80^hi^, Ly6C + (green gate in Figure 7) while monocyte 2 population contained cells that were CD11b^hi^, F4/80^lo^, Ly6C + (fuchsia gate in Figure 7). Resident macrophages (light blue gate in Figure 7) were not analyzed since there were not sufficient cells in the neonatal cell population for comparison. No difference was observed between the PBS and LPS treated groups for any marker (data not shown). Data are the mean ± SEM for 3 mice per group. Data were similar from two experiments, although different antibody concentrations were used in the second experiment (not shown). Statistical analysis was completed by two-way analysis of variance with Tukey’s multiple comparisons test. Significant age-specific differences are as indicated: *P < 0.05, **P < 0.01, ***P < 0.001.

To examine the functional state of these cell types, we examined cell surface markers related to cell activation: CD11a (integrin α L) and CD86; as well as those associated with inhibition of activation: CD200R (OX2R) and CD172a (SIRPα) [34-37]. Cells were analyzed at 6 hpi, that is, at the peak of cytokine mRNA expression. A significant difference was observed between neonatal and weanling mice in the mean fluorescent intensity (MFI) for these molecules (Figure 8), although the relative surface expression of these molecules was not altered by LPS stimulation (data not shown). Neonatal microglia had higher levels of CD11a and CD172a, but lower levels of CD86 (Figure 8). Neonatal monocytes had lower levels of CD11a than weanling monocytes, with varying levels of CD86, CD200R and CD172a depending on the subpopulation (Figure 8). Thus, there were distinct differences in the expression of cell surface regulatory markers between neonates and weanlings, but these varied substantially between the cell populations.

### SLAMF7 is expressed at high levels in neonatal microglia

**Figure 9:**
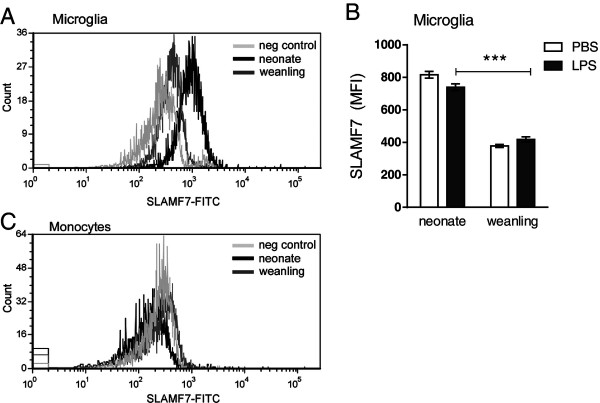
**SLAMF7 is strongly expressed by neonatal microglia.** Cells were isolated as described in Figure 7. **(A)** SLAMF7 expression on microglia cells (dark blue gate in Figure 7) from an LPS-inoculated neonatal mouse (black line) or weanling mouse (gray line). Light gray line is from a sample that did not contain the anti-SLAMF7 antibody (no antibody negative control). Plots shown are from a single mouse but are consistent with data from 2 to 4 mice per age. **(B)** Average MFI of SLAMF7 expression on microglia cells from PBS or LPS-treated mice showing a higher level of SLAMF7 on neonatal microglia. Data are the mean ± SEM for 3 LPS-treated mice per group at 6 hpi. Only two data points are plotted for PBS-treated controls and thus represent a range of data rather than SEM. Data are representative of two replicate experiments, although different antibody concentrations were used in the second experiment (not shown). ***P < 0.001 for direct comparison of LPS-treated groups **(C)** SLAMF7 expression was not detected on the monocyte populations (combined cells from green and fuchsia gates in Figure 7) when compared with the negative (no antibody) control (light gray line).

*Slamf7* was one of the markers whose mRNA expression was upregulated in whole brain tissue and in the myeloid/immune cells fraction from neonatal mice (Figure 5G, Figure 6C). SLAMF7 is often used as an activation marker for NK cells [31,32,38], however, we were unable to detect NK cells in the CNS by flow cytometry (data not shown). Although SLAMF7 was recently detected on activated human monocytes [39], expression in the CNS has not been reported. Analysis of SLAMF7 on the myeloid cells in the CNS demonstrated clear expression of SLAMF7 on neonatal microglia and lower levels of SLAMF7 on weanling microglia (Figure 9A), but was not upregulated following LPS stimulation (Figure 9B). SLAMF7 was not expressed at levels above background on the monocyte or macrophage population (Figure 9C, data not shown) for either PBS or LPS inoculated mice. Thus, SLAMF7 appears to be primarily expressed by neonatal microglia and may be a useful marker for identifying this population. The higher expression of SLAMF7, CD11a, F4/80 and CD172a on neonatal microglia compared with weanling microglia indicates a clear phenotype on these cells that correlates with the heightened inflammatory response in the CNS.

## Discussion

In the current study, we observed heightened responses to TLR stimulation in the CNS in neonatal mice compared with weanlings. This response was most notable with LPS, but was also observed with CpG-ODNs. Kinetic analysis of gene expression indicated that the increased cytokine response in neonates peaked at 6 hpi, which was associated with an increase in activation markers for myeloid cells in the CNS. Analysis of this cell population in the brains of neonatal and weanling mice indicated two primary differences in the myeloid population between these ages. The first was more macrophages in weanling mice and the second was increased expression of multiple regulatory molecules on neonatal microglia. Surprisingly, SLAMF7, a regulatory molecule on NK cells [32,38], was detected on neonatal, microglia and the mRNA expression of this molecule tightly correlated with cytokine induction.

The increased cytokine expression following TLR stimulation in neonates in the brain is opposite of the activation of monocytes/macrophages in the periphery. For example, neonatal mouse monocytes/macrophages have reduced cytokine responses to TLR4 stimulation compared with adults, with the exception of IL-10 production [40,41]. Similar age-related effects were also observed in studies with human monocytes/macrophages [12,16,18,19]. The primary reason for the differences in the periphery compared with the CNS may simply be the requirement for microglia in the CNS to be active in neonates for synaptic pruning and other developmental processes. A similar role in development has not been reported for peripheral monocytes. Thus, unlike adult animals in which the immune response in the CNS is considered to be more limited than in peripheral tissues, the CNS in neonatal mice may be more responsive to insult or injury and produce a more substantial inflammatory response. Since development of the neonatal mouse brain corresponds in several parameters with the second trimester development of the human brain [10,42], this time period in humans may be particularly sensitive to immune stimulation. However, since myeloid cells appear to be responsible for this increased inflammatory response, the inflammatory response in the human brain will most likely correlate with the timeframe in which these cells are most active during development, which may differ from that observed in mice.

The elevated response by the myeloid population in the neonatal CNS is surprising considering the lower level of *Tlr* mRNA expression and the higher levels of inhibitory molecules, including CD200R and CD172a (Figure 4, Figure 8). Deficiency in CD200R has been linked to increased inflammation in models of experimental autoimmune encephalitis (EAE) and Parkinson’s disease [43,44], while CD172a negatively regulates CD11b-mediated adhesion, migration and phagocytosis [35,45]. The higher expression of these receptors would predict a reduced responsiveness to TLR stimulation. However, the functional ability of CD172a and CD200R to inhibit myeloid cell activation may be dependent on many factors, including expression of their ligands, CD47 and CD200 respectively, on surrounding cells, as well as the expression of signaling molecules necessary to mediate inhibition of activation. Thus, the expression of these receptors may not be sufficient to diminish the inflammatory response to TLR stimulation in the neonatal brain.

The presence of SLAMF7 on neonatal microglia may be an additional negative regulator of microglia activation. SLAMF7 is a self-ligand and is involved in both cell activation as well as cell inhibition for NK cells [32]. However, analysis of activated human monocytes indicated that these cells did not express EAT-2, which is required for SLAMF7-induced activation [39]. SLAMF7 inhibited MAPK activity of monocytes [39], suggesting that SLAMF7 may function as a negative regulator of myeloid cell activation. It is possible that SLAMF7 may function in a manner similar to CD200R or CD172a and play a critical role in controlling the function of microglia and their interactions with neurons in the developing brain.

In addition to the elevated expression of negative regulators, neonatal microglia also had higher levels of integrins, including CD11a and CD11b. CD11b may be involved in TLR4 signaling by influencing the recruitment and degradation of intracellular TLR signaling adaptor proteins [46,47]. Therefore, elevated microglial CD11b levels could alter TLR4 signaling in neonatal microglia, resulting in the elevated cytokine responses observed in the CNS of neonatal mice.

The ratio of the Ly6C^hi^CD11b^hi^F4/80^lo^ monocytes (m2, fuschia gate) relative to the other three myeloid cell populations was higher in neonates than in weanling mice, although the relative number of cells m2 was comparable between age groups (Figure 7). The increased ratio of this cell population may indicate that this cell population is a contributor to the inflammatory response in neonates. Additionally, the reduced ratio of the other three cell populations could influence the inflammatory response if these cells had a regulatory role in limiting inflammation through the production of anti-inflammatory cytokines or inhibitory molecules. We did not observe any increase in IL-10 production in the weanling brain compared with the neonatal brain, indicating that these macrophages were not alternatively activated macrophages (data not shown). However, we cannot rule out localized production of inhibitory cytokines by these cells that would limit microglial or monocyte activation and cytokine production.

One of the few cytokines that was elevated in the weanling brain compared with the neonatal brain was *Csf2* (GMCSF) a cytokine known to be involved in the activation and maintenance of microglia and macrophages [48]. This induction of *Csf2* mRNA could be due to the higher percentages of the monocyte/macrophage populations in the weanling mice. Additionally, quiescent microglia may be programmed to produce more GMCSF upon stimulation to support cell activation and proliferation, while the neonatal amoeboid microglia are already in a heightened activation state.

Our studies indicate that myeloid cells are at least partially responsible for the increased proinflammatory response observed in neonates. However, other cell types including endothelia, oligodendroglia, neurons and astrocytes may also contribute to the differences in the cytokine production. Astrocytes have also been shown to produce cytokines in response to TLR stimulation, including TLR4 [28,49,50]. Astrocytes also undergo developmental changes in the first few weeks of birth, including morphological changes and gene expression [51,52], which may influence their response to TLR stimulation. Interestingly, *Gfap* mRNA expression, a marker of astrocyte activation, was lower in neonates compared with weanlings (Figure 5D). Thus, astrocytes may contribute to the neuroinflammatory response in both weanlings and neonates, but are unlikely to be a major source of the increased cytokine response in neonates.

## Conclusions

Although elevated levels of IL-6, IL-1β and TNF in the neonatal brain have been correlated with later cognitive disability and behavioral changes [7], few published studies directly compare the neuroinflammatory response of neonates with that of older animals. In our studies, we demonstrate that neonates have an altered neuroinflammatory response to TLR stimulation compared with older animals. The stronger response of myeloid cells to TLR stimulation following insult or injury to the CNS may have severe consequences in the neonate. Microglial production of soluble factors influence neuronal cell maturation and apoptosis and can affect neuroprogenitor proliferation and differentiation [20,22,53,54]. Stimulation via TLR4 alters the activation state of these cells as observed by the strong cytokine responses and *Nos2* mRNA expression (Figure 1, Figure 2). Thus, increased microglial activation may significantly alter brain development resulting in the long-term damage that has been associated with neonatal insults to the brain. Understanding the factors that regulate this distinct neuroinflammatory response will be important in preventing long-term damage following infection in the neonatal brain.

## Abbreviations

Actb: beta actin; BSA: bovine serum albumin; CCL: *CC* chemokine ligand; CNS: central nervous system; CpG-ODN: unmethylated CpG oligodeoxynucleotides; CSF2: colony stimulating factor 2 (granulocyte-macrophage); CXCL: CXC chemokine ligand; ELA2: neutrophil expressed elastase; GFAP: glial fibrillary acidic protein; GUSB: beta glucuronidase; HBSS: Hank’s balanced salt solution; IC: intracerebrally; IL: interleukin; ITGAX: integrin alpha X; LPS: lipopolysaccharide; MFI: mean fluorescent intensity; NK: natural killer; NOS2: inducible nitric oxide; PAMPs: Pathogen Associated Molecular Patterns; PBS: phosphate-buffered saline; PCR: polymerase chain reaction; RPL32: ribosomal protein L32; SLAMF7: signaling lymphocytic activation molecule family member 7; TLR: Toll-like Receptor; TNF: tumor necrosis factor.

## Competing interests

The authors declare that they have no competing interests.

## Authors’ contributions

LC and TW conducted the experiments including injections, removal of tissue, analysis of tissue by RT-PCR and processing of cells for flow cytometry. AC analyzed cells by flow cytometry. LC, BC and KP participated in conceiving and designing of the study. LC, BC and KP drafted the manuscript. All authors read and approved the final manuscript.
